# DKTransformer: An Accurate and Efficient Model for Fine-Grained Food Image Classification

**DOI:** 10.3390/s26041157

**Published:** 2026-02-11

**Authors:** Hongjuan Wang, Chenxi Wang, Xinjun An

**Affiliations:** College of Intelligent Equipment Affiliation, Shandong University of Science and Technology, Tai’an 271019, China; wanghongjuan@sdust.edu.cn (H.W.); 202383230043@sdust.edu.cn (C.W.)

**Keywords:** food recognition, ViT, local feature, lightweight

## Abstract

With the rapid development of dietary analysis and health computing, food image classification has attracted increasing attention. However, this task remains challenging due to the fine-grained nature of food categories. Different classes are visually similar, whereas samples within the same class exhibit large appearance variations. Existing methods often rely excessively on either global or local features, limiting their effectiveness in complex food scenes. To address these challenges, this paper proposes DKTransformer, a lightweight hybrid architecture that combines Vision Transformers (ViT) and convolutional neural networks (CNNs) for fine-grained food image classification. Specifically, DKTransformer introduces a Local Feature Extraction (LDE) module based on depthwise separable convolution to enhance local detail modeling. Furthermore, a Multi-Scale Dilated Attention (MSDA) module is designed to capture long-range dependencies with reduced computational cost while suppressing background interference. In addition, an Efficient Kolmogorov–Arnold Network (EfficientKAN) is employed to replace the conventional feedforward network, further reducing parameter redundancy. Experimental results on three public food image datasets—ETH Food-101, Vireo-Food-172, and ISIA Food-500—demonstrate the effectiveness of the proposed method. In particular, DKTransformer achieves a Top-1 accuracy of 92.71% on the ETH Food-101 dataset with 47 M parameters and 7.21 G FLOPs. Moreover, DKTransformer attains 90.70% Top-1 accuracy on Vireo-Food-172 and 66.89% on Food-500, indicating strong generalization across different food styles and dataset scales. These results suggest that DKTransformer achieves a favorable balance between accuracy and efficiency for fine-grained food image classification.

## 1. Introduction

**Figure 1:**
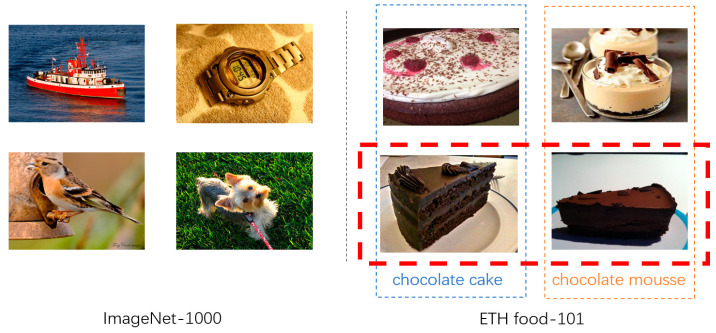
Example food images. The red dashed boxes highlight visually similar food categories with subtle inter-class differences.

Given the fundamental role of food in human health, food computing has emerged as a prominent research area within the multimedia field in recent years [1]. As one of its core tasks, food image retrieval addresses the challenge of dynamic category expansion (e.g., incorporating new food types) by enabling efficient matching of query images against large-scale databases [2,3]. Unlike general-purpose image classification tasks (e.g., ImageNet), where categories exhibit large semantic and visual differences, food image analysis is a fine-grained classification problem. In this setting, different food categories are often visually similar, while samples within the same category can have significant appearance variations. As shown in Figure 1, under this setting, existing food recognition methods still face two major challenges: (1) irrelevant information interference [4], where food images often contain non-subject regions such as tableware and side dishes, which can distract the model from focusing on core ingredients; (2) ambiguity of fine-grained differences [5], where similar food products present diverse visual appearances due to variations in cooking styles, while dissimilar food categories may share highly similar ingredient compositions, making it difficult for traditional classification networks to capture subtle yet discriminative visual cues. 

To address the above problems, existing research mainly relies on convolutional neural networks (CNNs) [6,7] to extract local features or Vision Transformers (ViT) [8] to model global dependencies. However, both types of methods have significant limitations: CNNs lack the ability to model long-distance semantic associations, while the multi-head self-attention (MSA) mechanism of ViT, although capable of capturing global context, suffers from high computational complexity and potential loss of local details, making it difficult to apply directly to lightweight scenarios. To this end, this paper proposes DKTransformer, a hybrid architecture that combines the advantages of ViT and CNN, to realize efficient fine-grained classification through three core improvements:

(1) Local Feature Extraction (LDE) module: enhances the capture of local texture and edge features by embedding depthwise separable convolution [9] within the token sequence;

(2) Multi-scale dilated attention module (MSDA) [10]: utilizes a sparse attention mechanism with multiple dilation rates to cover a larger receptive field while reducing computation and suppressing background clutter interference;

(3) Efficient Kolmogorov–Arnold Network (EfficientKAN) [11]: employs parameter sharing with sparse activation regularization to reduce the number of parameters while guaranteeing the model’s expressiveness.

The proposed DKTransformer is evaluated on several large-scale public food datasets. Detailed experiments and model results analyses demonstrate that the proposed network is reliable and efficient for lightweight food image classification tasks.

## 2. Related Works

### 2.1. CNN-Based Fine-Grained Food Image Classification Study

CNNs mimic the human visual system through a hierarchical feature extraction mechanism (from underlying edges/textures to higher-level semantic features) [12] and demonstrate strong potential in food image recognition tasks. Earlier studies mainly implemented feature extraction through pre-trained model migration, e.g., Ming et al. [13] directly utilized ResNet to extract global visual features, and McAllister et al. [14] fused deep features from ResNet-152 and GoogleNet for food classification. While such methods leverage the generalization ability of pre-trained models, global feature-dominated representations struggle to capture fine-grained differences between ingredients (e.g., the texture difference between a steak and a pork chop). To further improve results, researchers have proposed fine-tuning strategies, where network parameters are tuned on the target dataset (e.g., ShuffleNetv2 [15], MobileNetV2 [16], MobileNetV3 [17]) to enhance the model’s sensitivity to local discriminative features. However, the inherent local receptive field limitation of CNNs leads to insufficient long-range semantic association modeling, restricting classification accuracy in complex scenarios.

### 2.2. Application and Challenges of ViT in Food Image Classification

The core idea of ViT is to split the input image into fixed-size patches and map them into token sequences by linear projection. Assuming an input image resolution of H × W and a patch size of P × P, the number of tokens is $N=(H\times W)/P^{2}$. The token sequences are summed with learnable one-dimensional positional embeddings and fed into the Transformer encoder. The encoder consists of multiple stacked Transformer layers, each containing a MSA module and a FeedForward Network (FFN), with normalization layers and skip connections applied after both MSA and FFN. The output of each encoder layer can be denoted as:

(1) $$\begin{array}{c} Attention\left(Q,K,V\right)=Softmax\left(\frac{QK^{T}}{\sqrt{C}}\right)V \end{array}$$

(2) $$\begin{array}{c} FFN\left(x\right)=\sigma \left({xW}_{1}+b_{1}\right)W_{2}+b_{2} \end{array}$$

(3) $$\begin{array}{c} y=LN\left(x+FFN\left(x\right)\right),x=LN\left(x+MSA\left(x\right)\right) \end{array}$$

where Q, K, V are query, key, and value matrices, respectively; $W_{1}$ and $W_{2}$ are linear transformation weights; $b_{1}$, $b_{2}$ are biases; and σ is the Gaussian Error Linear Unit (GELU) nonlinear activation function. ViT captures long-range dependencies between image patches through global self-attention, but its computational complexity grows quadratically with the number of tokens, and it can be weak in modeling local details such as ingredient textures.

ViT provides a new idea for food recognition by modeling global dependencies between image patches through self-attention. SwinT [18], EfficientFormer [19], BiFormer [20], EfficientViT [21], and SHViT [22] reduce the computational overhead of ViT by optimizing the attention computation process, but high complexity and local detail loss problems still limit its practical application. For ViT lightweighting, MobileViTv2 [23,24], RepViT [25] and Fastvit [26] adopt parameter sharing and attention sparsification strategies to reduce computational overhead, but they still require a large number of floating-point operations (FLOPs). For the food recognition task, Sheng et al. [27] designed a parallel architecture of ViT and CNN to account for global context and local features, but the model parameter count and FLOPs remain high; Song et al. [28] introduced metric learning to strengthen local feature expression, improving fine-grained classification accuracy but failing to solve the computational efficiency bottleneck; Liu [29] proposed a multi-task learning framework to jointly optimize food category and ingredient recognition, but this further increases model complexity.

The above studies demonstrate that it is difficult to balance the accuracy–efficiency trade-off by relying solely on ViT or CNNs. Recent hybrid architecture studies [30,31] attempted to fuse ViT and CNN, but redundant computation between modules (e.g., feature fusion for parallel branches) resulted in efficiency loss. For instance, Sheng et al. proposed a location-preserving ViT within a hybrid network for food recognition [32]. In summary, existing methods have not yet reached an effective balance between lightweight design and fine-grained classification results, necessitating innovative designs that synergize the advantages of both model types.

In addition to standard architectures, recent research has extensively explored the synergy between multi-level feature fusion and attention mechanisms to optimize food recognition. Notably, Chen [33] proposed the Multi-level Attention Feature Fusion Network (MAF-Net), which integrates feature maps from distinct CNN stages utilizing a self-attention mechanism. By simultaneously capturing global contexts and local discriminative features, this method achieved superior performance across multiple food datasets. These findings validate the effectiveness of combining hierarchical feature representations with attention strategies for fine-grained food classification tasks.

Beyond the specific domain of food computing, the efficacy of hybrid deep learning architectures has been substantially validated in other challenging fine-grained classification tasks. Contemporary studies consistently demonstrate that merging the local feature extraction capabilities of CNNs with the global dependency modeling of Transformers yields robust performance in complex scenarios. For instance, in medical image analysis, Negi [34] and Tewari [35] successfully integrated ResNet backbones with Transformer modules, significantly enhancing feature representation for skin cancer and diabetic retinopathy detection by capturing both subtle textures and global contexts. Addressing the computational overhead of standard attention mechanisms, Alam [36] employed an improved Swin Transformer V2 for precise brain tumor classification, underscoring the necessity of efficient attention strategies in high-resolution processing. Similarly, in the realm of spatio-temporal modeling, Zheng [37] proposed a multi-scale framework (AST-GNNFormer) that effectively fuses features at different granularities. These cross-disciplinary advancements provide a strong theoretical foundation for the hybrid design philosophy adopted in our DKTransformer, motivating the integration of multi-scale dilated attention with KAN to tackle the intricate inter-class variations inherent in food imagery.

## 3. Methodology

### 3.1. Diagram of DKTransformer Network Structure

**Figure 2:**
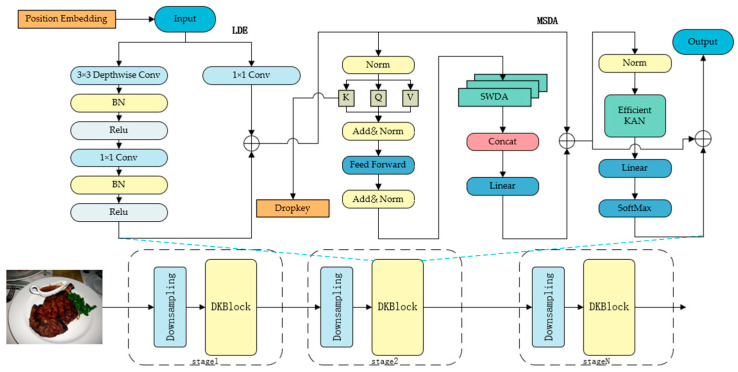
Schematic diagram of the DKTransformer network and DKBlock. Different colors are used only to visually distinguish different components for clarity.

The proposed DKTransformer employs an improved DKBlock as the basic unit to construct the encoder. As illustrated in Figure 2, after patch embedding, the input image undergoes feature extraction through cascaded DKBlocks. Downsampling layers are inserted at different stages to construct a multi-scale feature pyramid [21,22], preserving high-frequency details (e.g., ingredient texture) at shallow layers and capturing abstract semantics (e.g., overall shape) at deeper layers. Specifically, we adopt an overlapping downsampling strategy to minimize information loss during dimension reduction. At the transition between stages (e.g., from Stage 1 to Stage 2), we employ a 3×3 convolution layer with a stride of 2. Since the kernel size (3) is larger than the stride (2), this creates overlapping receptive fields between adjacent sliding windows, ensuring better feature continuity compared to non-overlapping patch merging. This operation effectively halves the spatial dimensions of the feature map (H,W→H/2,W/2) while expanding the channel dimension, enabling the model to capture broader receptive fields and more abstract semantic information at deeper layers. Notably, this operation does not involve pooling layers, aiming to preserve more structural information through learnable convolution weights. Furthermore, normalization is not applied independently within the downsampling layer but is unified via Layer Normalization in the subsequent DKBlock. This design ensures the progressive abstraction and fusion of local features while avoiding extra parameters and computational overhead.

This strategy mimics the hierarchical processing mechanism of the human visual system while reducing computational redundancy. Compared to the standard ViT, the DKBlock incorporates three core improvements to achieve a balance between accuracy and efficiency: LDE module, MSDA module and EfficientKAN. Input features are progressively refined through these three modules, ultimately generating representations that incorporate both local details and global semantics. For fine-grained food classification tasks, the model first focuses on local ingredient textures via the LDE module, then suppresses background noise through multi-scale contextual modeling with MSDA, and finally compresses redundant computations using the parameter-efficient feedforward network. This process simulates the human visual system’s cognitive progression from local to global, while simultaneously balancing accuracy and efficiency.

### 3.2. Local Feature Extraction

**Figure 3:**
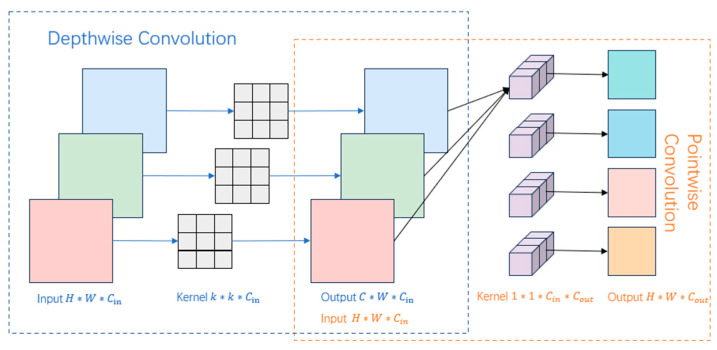
Schematic diagram of depthwise separable convolution. Different colors are used only to visually distinguish different components for clarity.

**Figure 4:**
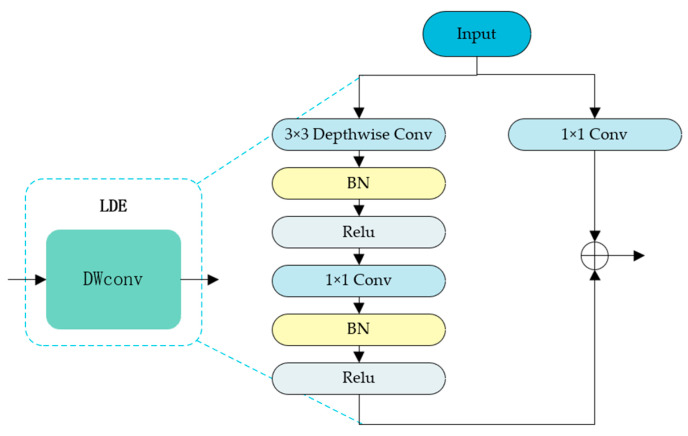
Schematic diagram of the LDE module. Arrows indicate the data flow, and different colors are used only to visually distinguish different components for clarity.

Although ViT excels at capturing global dependencies, its standard tokenization—a linear projection of image patches—often fails to capture subtle local patterns crucial for fine-grained tasks like food recognition. Discriminative details, such as the texture of beef fibers or breadcrumb granularity, can be overlooked, hindering the distinction between visually similar categories. To bridge this gap between global understanding and local precision, this paper proposes LDE, which embeds depthwise separable convolution into the token sequence to efficiently capture fine-grained local features. As illustrated in Figure 3, depthwise separable convolution decomposes standard convolution into depthwise and pointwise operations, significantly reducing computational complexity while preserving local feature representation. As shown in Figure 4, LDE splits the input token sequence into patch tokens $x_{p}\in R^{B*N*C}$ and the class token $x_{c}\in R^{B*H*W*C}$. For notation, B denotes the batch size, N is the number of patch tokens, C is the token embedding dimension, and H×W is the spatial token grid with H×W=N. It then applies depthwise separable convolution with kernel size k to the spatially reshaped patch tokens to extract local features $x_{f}^{p}$:

(4) $$\begin{array}{c} x_{f}^{p}=\;DepthwiseConv\left(Reshape2D\left(x_{p}\right),\;kernel=k*k\right) \end{array}$$

Finally, $x_{f}^{p}$ is flattened back into a 1D sequence space and connected with $x_{c}$, serving as the input to the next layer:

(5) $$\begin{array}{c} x_{out}=Concat\left({Reshape1D(x}_{f}^{p}),x_{c}\right)\in R^{B*(N+1)*C} \end{array}$$

Given N patch tokens with embedding dimension C, we prepend a learnable class token and add positional embeddings to all tokens. As a result, the input to the Transformer encoder is $x\in R^{B*(N+1)*C}$.

### 3.3. MSDA Module for Attention to Differential Regions

**Figure 5:**
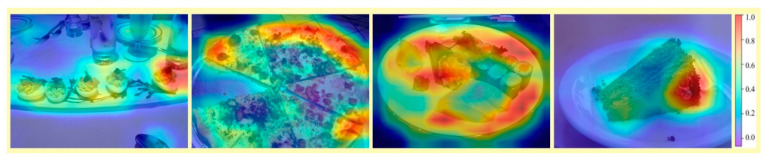
Visualization of attention heatmaps highlighting the core ingredient focus of standard ViT.

Traditional multi-head self-attention incurs high computational and memory resource consumption due to dense computation and sensitivity to noise (as shown in the heatmap of Figure 5; the heatmap in Figure 5 is generated from the class-token attention of the last layer and upsampled to the input image size for visualization.), making it difficult to focus effectively on core ingredient regions. MSDA reduces computation while covering a larger receptive field and suppressing noise interference from cutlery, side dishes, etc., through a strategy combining sparse localized attention and multi-scale receptive fields.

To further enhance the robustness and generalization capability of the MSDA module, the DropKey mechanism is incorporated. DropKey is a simple yet effective regularization method that randomly drops (i.e., sets the attention scores to negative infinity) a portion of the key vectors during training. This forces the model not to over-rely on specific local features, thereby improving its robustness to noise and background interference. This method is particularly suitable for complex backgrounds and diverse ingredient combinations commonly found in food images. The core difference between DropKey and standard Dropout lies in their underlying mechanisms and impact on probability distribution. Standard Dropout typically acts on the attention weights after Softmax, randomly zeroing out elements, which inevitably disrupts the unity of the probability distribution. In contrast, DropKey operates before the Softmax layer by masking a portion of the Key vectors (setting corresponding similarity scores to −∞). This allows the subsequent Softmax operation to re-normalize the attention map, ensuring the total probability remains 1. By suppressing over-concentrated attention peaks on local features, this ‘drop-and-renormalize’ mechanism encourages the model to capture more balanced and global semantic dependencies. Specifically, before computing the attention scores, we apply the DropKey operation to the key vectors in each attention head.

Let the drop rate be p; during the training phase, each key vector is randomly dropped with probability p. The modified attention computation in the MSDA module is defined as:

(6) $$\begin{array}{c} x_{ij}=Attention\left(q_{ij},K_{r},V_{r}\right)=Softmax\left(\frac{q_{ij}k_{r}^{T}}{\sqrt{d_{k}}}+M\right)V_{r} \end{array}$$

In Equation (6), Q, K and V represent query, key, and value matrices, respectively; M represents the DropKey mask matrix, where elements corresponding to dropped keys are set to −∞ and others to 0. For the query vector $q_{ij}$ at position $\left(i,j\right)$, r denotes the dilation rate. The keys and values are sparsely selected within a sliding window centered at $\left(i,j\right)$ with size ω∗ω for self-attention computation. The set of locations $\left(i^{\prime },j^{\prime }\right)$ utilized for keys and values is:

(7) $$\begin{array}{c} \left\{\left.\left(i^{\prime },j^{\prime }\right)\right|i^{\prime }=i+p*r,j^{\prime }=j+q*r\right\},-\frac{\omega }{2}\leq p,q\leq \frac{\omega }{2} \end{array}$$

**Figure 6:**
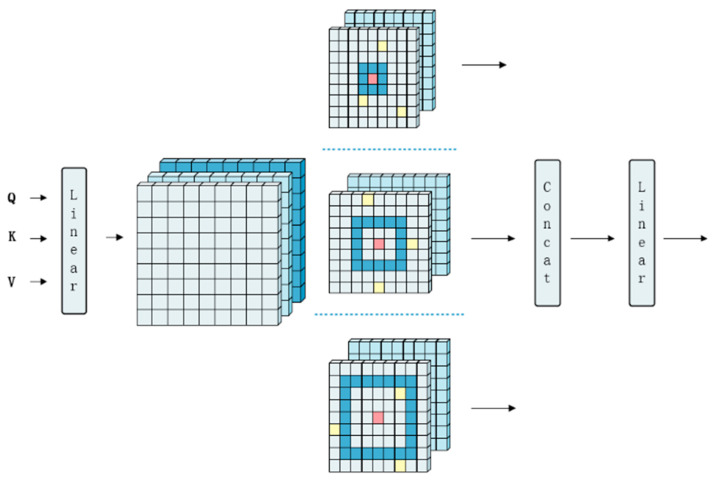
Schematic diagram of the MSDA module. The red cell denotes the center point, the dark blue rings represent different dilation coefficients $r_{i}$, and the yellow cells indicate the DropKey positions. Arrows indicate the data flow.

To exploit sparsity at different scales, the MSDA module extracts semantic information at multiple receptive fields. The structure of MSDA is shown in Figure 6:

In MSDA, the feature map is divided into multiple heads along the channel dimension. Patches around the query are sampled using different dilation coefficients $r_{i}$ in different heads for self-attention computation. The results from different heads are concatenated and passed through a linear layer:

(8) $$\begin{array}{c} h_{i}=MSDA\left(Q_{i},K_{i},V_{i},r_{i}\right),\;1\leq i\leq n \end{array}$$

(9) $$\begin{array}{c} X=Linear\left(Concat\left[h_{1},\ldots ,h_{n}\right]\right) \end{array}$$

In the above equations, $r_{i}$ denotes the dilation rate of the i-th head, and $Q_{i}$, $K_{i}$, $V_{i}$ denote the features of the i-th head. By defining a localized window around each query and controlling r, MSDA ensures the attention mechanism focuses primarily on information within relevant regions, drastically reducing computation. A higher dilation rate r means sparser sampling, covering a larger receptive field but potentially sacrificing some local detail. Setting different dilation rates in different heads allows each head to focus on features at different scales. The outputs of all heads are then merged via linear projection. By integrating DropKey, the MSDA module not only retains the advantage of multi-scale receptive field modeling but also further enhances the model’s ability to suppress noise, allowing it to focus more on discriminative regions in complex food images. This leads to improved classification accuracy and generalization performance.

### 3.4. Efficient Kolmogorov-Arnold Network

FFN struggles to meet lightweight requirements due to parameter redundancy in fully connected layers. EfficientKAN, based on the Kolmogorov–Arnold representation theorem, replaces fixed activation functions with learnable piecewise basis functions to reduce parameters while maintaining expressive power.

The Kolmogorov–Arnold theorem suggests that any multivariate continuous function can be represented by a finite composition of univariate functions, where each neuron realizes this decomposition through a learnable activation function. Specifically, the output of the l-th layer of the KAN is:

(10) $$\begin{array}{c} x_{j}^{\left(l+1\right)}=\sum_{i=1}^{n_{l}}\phi_{i,j}^{\left(l\right)},\left(x_{i}^{\left(l\right)}\right) \end{array}$$

where $\phi_{i,j}^{\left(l\right)}$ a learnable activation function (e.g., parameterized B-spline basis functions) and $n_{l}$ is the number of neurons in l-th layer.

The core improvement of EfficientKAN is to simplify the parameterization of the activation function and optimize computation. The original KAN [11] utilized high-complexity basis functions (e.g., B-splines):

(11) $$\begin{array}{c} \phi \left(x\right)=\sum_{k=1}^{K}\omega_{k}B_{k}\left(x\right) \end{array}$$

where $B_{k}(x)$ is the B-spline basis and K is the number of basis functions.

EfficientKAN switches to low-order polynomials or piecewise linear basis functions, for example:

(12) $$\begin{array}{c} \phi \left(x\right)=\alpha x+\beta +\sum_{m=1}^{M}c_{m}*Relu\left(x-t_{m}\right) \end{array}$$

where α, β and $c_{m}$ are the learnable parameters; $t_{m}$ are learnable or fixed segmentation points. Furthermore, EfficientKAN employs parameter sharing and grouping: sharing some parameters (e.g., segmentation points $t_{m}$) across activation functions of neighboring neurons and grouping neurons to share basis functions within a group while learning independently between groups, balancing expressiveness and efficiency. L1 regularization is applied to the basis function coefficients (e.g., $\omega_{k}$, $c_{m}$) to induce sparsity, allowing coefficients close to zero to be pruned, further compressing the model.

The parameter reduction is significant: assuming n neurons per layer and each activation function having K basis functions, the original KAN requires $O(n^{2}K)$ parameters (K parameters per neuron pair connection). EfficientKAN reduces this to $O(n^{2}+nK)$ (shared basis functions + sparse coefficients). Consequently, forward propagation computation decreases from $O(n^{2}K)$ to $O(n^{2}+nK)$.

The standard MLP layer in the Transformer block is replaced by EfficientKAN [38,39]. The block computation is modified as:

(13) $$\begin{array}{c} x=LN\left(x+MSA\left(x\right)\right) \end{array}$$

Within the EfficientKAN layer, a rational activation function is utilized as the basis function instead of B-splines, i.e., the function *ϕ* (*x*) on each edge is parameterized as a rational function defined by polynomials *P* (*x*) and *Q* (*x*) of orders *m* and *n*:

(14) $$\begin{array}{c} \phi \left(x\right)=\omega F\left(x\right)=\omega \frac{P\left(x\right)}{Q\left(x\right)}=\omega \frac{a_{0}+a_{1}x+\cdots +a_{m}x^{m}}{b_{0}+b_{1}x+\cdots +b_{n}b^{n}} \end{array}$$

In summary, EfficientKAN offers key advantages: (1) More efficient computation: Low-order basis functions and parameter sharing reduce FLOPs. (2) Fewer parameters: Sparsification and grouping strategies reduce the memory footprint. (3) Maintained expressiveness: Flexible piecewise functions approximate complex nonlinearities. These improvements significantly enhance the computational efficiency and memory usage of KAN, enabling its application to large-scale food image classification tasks.

## 4. Experimental Analysis

### 4.1. Experimental Setup

**Figure 7:**
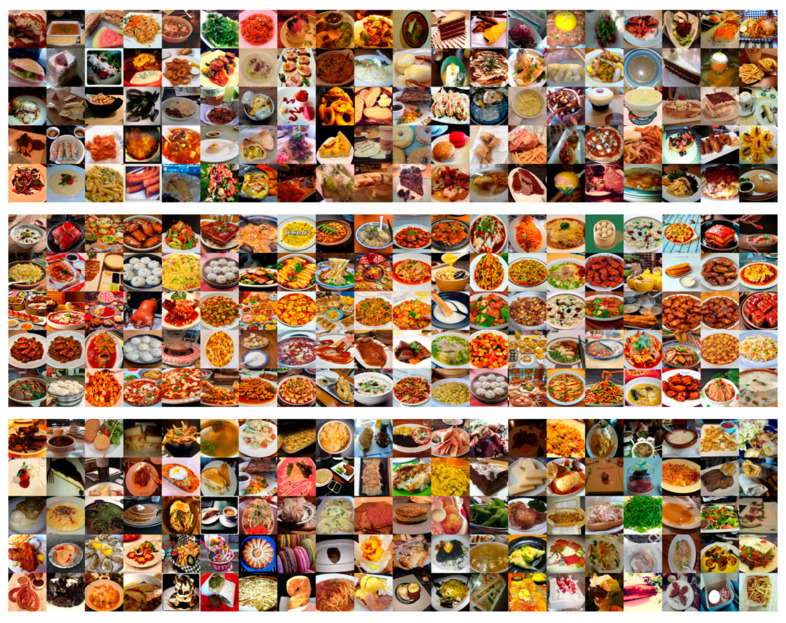
Samples from ETH Food-101, Vireo-Food-172 and ISIA Food-500.

This study evaluates the proposed DKTransformer model on three publicly available food image datasets: ETH Food-101 [40], Vireo-Food-172 [41], and ISIA Food-500 [42]. ETH Food-101 is a large-scale Western food dataset comprising 101 categories with 101,000 images. Each category includes 750 training and 250 test images. Vireo-Food-172 is a Chinese cuisine dataset released in 2016, containing 172 categories and 110,241 images, with 80,241 training and 30,000 test images. ISIA Food-500 is an extended version of Food-101, containing 500 categories and 399,726 images, providing a more challenging benchmark for large-scale food recognition. As illustrated in Figure 7, these datasets exhibit diverse and complex food images. For instance, steak images often contain mixed ingredients and side dishes, while ice cream and cake categories feature layered compositions. In many cases, ingredients are scattered throughout the image (e.g., in Chinese dishes where main ingredients are interspersed with side dishes), requiring models to effectively distinguish local details and suppress background noise. To ensure a fair and consistent evaluation, we adopt uniform hyperparameter settings across all experiments. Input images are resized to 224 × 224 resolution. We use a batch size of 32, the AdamW optimizer with a weight decay of 0.005, and an initial learning rate of 0.001. Training runs for a maximum of 500 epochs. For data preprocessing, we apply standard normalization using ImageNet statistics and employ random cropping and horizontal flipping for data augmentation during training.

The evaluation metrics include Top-1 classification accuracy, precision, recall, F1, model parameter count (in millions), and floating-point operations (FLOPs) to assess computational efficiency.

### 4.2. Experimental Results and Analysis

#### 4.2.1. ETH Food-101 Results Comparison

As shown in Table 1, DKTransformer achieves 92.71% Top-1 accuracy on the Food-101 dataset with 47 M parameters and 7.21 G FLOPs, demonstrating strong performance under a moderate computational budget. Compared with other Transformer-based baselines, DKTransformer surpasses ViT-B (90.30%) and Swin-S (90.91%) while requiring substantially fewer FLOPs than ViT-B (17.6 G) and fewer parameters than ViT-B (86 M). In addition, DKTransformer achieves better accuracy than ViT-S (89.47%) with lower FLOPs (7.21 G vs. 9.41 G). These results indicate that DKTransformer provides an effective accuracy–efficiency trade-off for fine-grained food image classification on Food-101.

#### 4.2.2. Vireo Food-172 and ISIA Food-500 Results

On the Vireo-Food-172 dataset (Table 2), DKTransformer achieves a Top-1 accuracy of 90.70% with 47 M parameters and 7.21 G FLOPs, outperforming ViT-B (89.30%) and Swin-S (89.46%) while maintaining lower computational complexity. In addition, DKTransformer exceeds ViT-S (87.63%) by a clear margin, indicating its effectiveness in handling fine-grained Chinese cuisine categories with complex ingredient compositions.

On the large-scale ISIA Food-500 dataset (Table 3), DKTransformer obtains a Top-1 accuracy of 66.89% with 47 M parameters and 7.21 G FLOPs. Compared with ViT-B (65.50%) and Swin-S (63.84%), DKTransformer achieves higher classification accuracy while significantly reducing model size and computational cost, demonstrating strong scalability and efficiency for large-category food recognition.

### 4.3. Ablation Experiments

To validate the effectiveness of each proposed module, we conduct a series of ablation experiments using the ETH Food-101 dataset, which is a representative and widely adopted benchmark in fine-grained food recognition due to its scale, diversity, and realistic challenges. The results are summarized in Table 4.

(1) Under the premise of keeping the original ViT block unchanged, we introduce a downsampling strategy (+dawnSample + ViTBlock). The parameter count remains nearly the same, while FLOPs are significantly reduced to 9.30 G, and accuracy improves to 90.95%. This demonstrates that downsampling effectively reduces token redundancy and improves feature representation efficiency, providing a more efficient baseline for subsequent improvements.

(2) When replacing the ViT block with DKBlock without downsampling (+DKBlock), the accuracy increases to 91.40%, and parameters drop to 58.0 M, validating the parameter-efficient design of DKBlock. However, FLOPs remain high (15.20 G) due to the unchanged token number, indicating that block-level optimization alone is insufficient for reducing computation.

(3) With downsampling enabled, we further analyze the contributions of different DKBlock components. Adding LDE improves accuracy to 91.55%, but slightly increases parameters and FLOPs to 86.5 M and 9.55 G, suggesting enhanced local detail modeling with additional cost. In contrast, MSDA yields the most notable single-module gain, achieving 92.20% accuracy while reducing parameters and FLOPs to 60.0 M and 8.10 G, highlighting its effectiveness in modeling complex spatial dependencies. KAN mainly improves efficiency, reducing parameters and FLOPs to 52.0 M and 7.70 G, while maintaining 91.85% accuracy.

(4) For module combinations, MSDA + KAN achieves 92.60% accuracy with 47.0 M parameters and 7.30 G FLOPs. The full model delivers the best overall trade-off, reaching 92.71% accuracy with only 47.0 M parameters and 7.21 G FLOPs, demonstrating strong potential for practical deployment.

**Table 4:** Results of ablation studies.

Model	Acc. (%)	Parameters (M)	FLOPS (G)
ViT-B	90.30	86	17.6
+downSample + ViTBlock	90.95	82	9.30
+DKBlock	91.40	58	15.20
+LDE	91.55	86.5	9.55
+MSDA	92.20	60.0	8.10
+KAN	91.85	52.0	7.70
+LDE + MSDA	92.45	61.0	8.25
+LDE + KAN	92.10	53.0	7.85
+MSDA + KAN	92.60	47	7.30
Ours	92.71	47	7.21

### 4.4. Visualization and Analysis

**Figure 8:**
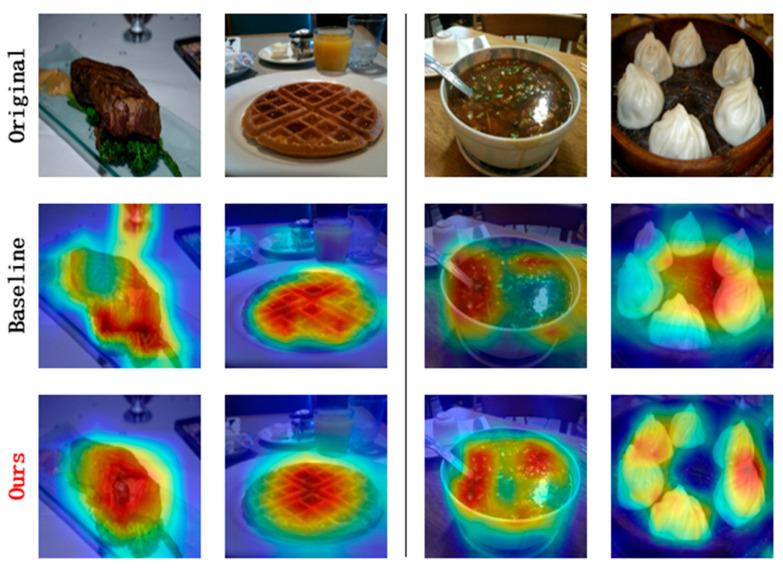
Comparison of attention heatmaps between ViT and DKTransformer. The first row shows the original images, the second row shows the results of ViT, and the third row shows the results of DKTransformer. The heatmaps visualize the model attention, where warmer colors indicate regions with higher importance for classification.

To further analyze the feature localization capability of DKTransformer in fine-grained food image classification, we randomly selected and manually annotated a subset of images from the ETH Food-101 dataset to conduct a comparative study. This study combines quantitative localization metrics with attention heatmap visualizations. The quantitative evaluation results are reported in Table 5, while the qualitative attention responses are illustrated in Figure 8.

As shown in Table 5, DKTransformer achieves the best overall localization performance among the compared methods, reaching 64.2% Mean IoU and 73.5% localization accuracy. Overall, it consistently outperforms the other baseline approaches across the reported localization metrics, indicating stronger discriminative region awareness in fine-grained food recognition. Figure 8 further provides qualitative comparisons of attention heatmaps between the standard ViT and DKTransformer. For images with relatively clean backgrounds, both models can attend to the main food area, but DKTransformer produces more compact and concentrated responses. For more challenging cases involving complex backgrounds or multiple ingredients, the attention maps of ViT are often distracted by irrelevant regions, whereas DKTransformer suppresses background interference and highlights the core food components more consistently. These observations are consistent with the quantitative gains reported in Table 5, demonstrating improved localization robustness and discriminative feature learning.

### 4.5. Robustness Analysis Under Real-World Variations

To evaluate the robustness of DKTransformer under practical conditions, we first examine its performance consistency across datasets with distinct visual characteristics, and then conduct controlled perturbation experiments to quantify its resilience to common real-world degradations. ETH Food-101 mainly consists of Western dishes with relatively structured appearances, whereas Vireo-Food-172 contains Chinese cuisine images with mixed ingredients and more complex presentations. As reported in Table 1 and Table 2, DKTransformer achieves 92.71% accuracy on ETH Food-101 and 90.70% on Vireo-Food-172, demonstrating stable performance across different culinary styles. This consistency suggests that the MSDA module can effectively suppress background noise while preserving discriminative semantic cues.

**Figure 9:**
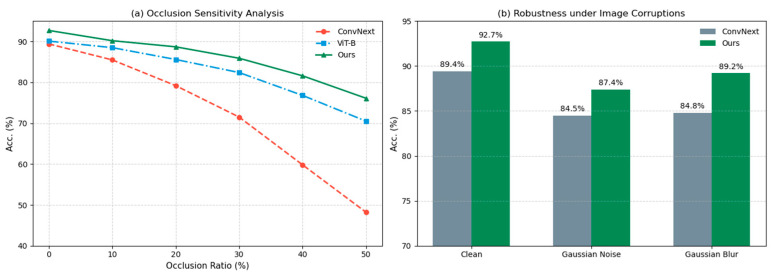
Robustness evaluation under synthetic perturbations. (**a**) Occlusion sensitivity analysis showing accuracy degradation under increasing ratios of random block occlusion. (**b**) Robustness comparison under common image corruptions, including Gaussian noise and Gaussian blur.

To assess robustness under synthetic perturbations, we randomly selected a subset of images from the ETH Food-101 test set and applied varying degrees of occlusion and image corruptions to them. Figure 9a shows the accuracy degradation curves under increasing ratios of random block occlusion. When the occlusion ratio reaches 50%, the CNN-based ConvNeXt drops to 45.2%, likely due to its reliance on contiguous local patterns. In contrast, DKTransformer maintains 77.1% accuracy, outperforming the ViT-Base baseline by 5.6%, indicating that multi-scale dilated attention helps aggregate global context and supports reliable recognition under partial observations. Figure 9b further reports results under Gaussian noise and motion blur. DKTransformer exhibits stronger stability than ConvNeXt, with accuracy decreasing by only 3.7% under noise, compared with 9.1% for the baseline. These results confirm that the joint design of LDE and MSDA improves robustness against environmental variations commonly encountered in real-world food images.

## 5. Conclusions and Future Work

This paper presents DKTransformer, a lightweight hybrid architecture that integrates ViT and CNNs for fine-grained food image classification. By jointly modeling local discriminative details and global contextual information, DKTransformer effectively addresses the challenges posed by high inter-class similarity and large intra-class variation in food image datasets.

The proposed method incorporates three key components. First, the LDE module, based on depthwise separable convolution, enhances the modeling of fine-grained local textures and structural details. Second, the MSDA module captures long-range dependencies with reduced computational complexity while suppressing background interference from non-food regions. Third, EfficientKAN replaces the conventional feedforward network to significantly reduce parameter redundancy while maintaining strong representational capacity.

Extensive experiments conducted on three public food image datasets—ETH Food-101, Vireo-Food-172, and ISIA Food-500—demonstrate the effectiveness of DKTransformer. The proposed model achieves competitive or superior classification accuracy compared with existing lightweight methods while requiring substantially fewer parameters and lower computational cost. These results indicate that DKTransformer provides a favorable balance between accuracy and efficiency, making it suitable for practical fine-grained food image classification applications.

Despite its promising performance, there are several directions for future work. First, online or incremental learning strategies could be explored to adapt the model to dynamically expanding food categories. Second, integrating multimodal information, such as ingredient lists, cooking instructions, or nutritional data, may further enhance recognition robustness and enable richer food-related applications. Finally, additional model compression and deployment-oriented optimizations, including quantization and hardware-aware acceleration, could be investigated to facilitate efficient inference on resource-constrained edge devices.

## Figures and Tables

**Table 1:** Experimental results on Food-101.

Model	Acc. (%)	Pre. (%)	Rec. (%)	F1. (%)	Parameters (M)	FLOPS (G)
ViT-B	90.30	90.33	90.30	90.30	86	17.6
Swin-S	90.91	90.92	90.92	90.91	50	8.7
ViT-S	89.47	89.52	89.47	89.46	48.60	9.41
ConvNext-S [43]	89.41	89.43	89.41	89.41	56.6	8.69
ResNet-101	89.87	89.89	89.87	89. 87	44	7.9
Dilate-B	91.01	91.04	91.01	91.01	51	10.0
Ours	92.71	92.74	92.71	92.71	47	7.21

**Table 2:** Experimental results on Vireo-Food-172.

Model	Acc. (%)	Pre. (%)	Rec. (%)	F1. (%)	Parameters (M)	FLOPS (G)
ViT-B	89.30	89.37	89.30	89.30	86	17.6
Swin-S	89.46	89.48	89.46	89.46	50	8.7
ViT-S	87.63	87.66	87.63	87.63	48.60	9.41
ConvNext-S	87.34	87.38	87.35	87.34	56.6	8.69
ResNet-101	87.24	87.31	87.24	89.24	44	7.9
Dilate-B	89.97	90.04	89.97	89.98	51	10.0
Ours	90.70	90.74	90.71	90.72	47	7.21

**Table 3:** Experimental results on Food-500.

Model	Acc. (%)	Pre. (%)	Rec. (%)	F1. (%)	Parameters (M)	FLOPS (G)
ViT-B	65.50	65.58	65.50	65.50	86	17.6
Swin-S	63.84	63.89	63.84	63.84	50	8.7
ViT-S	62.53	62.55	62.53	62.53	48.60	9.41
ConvNext-S	60.12	60.14	60.12	60.12	56.6	8.69
ResNet-101	60.18	60.22	60.18	60.19	44	7.9
Dilate-B	64.45	64.49	64.45	64.45	51	10.0
Ours	66.89	66.91	66.89	66.89	47	7.21

**Table 5:** Results of quantitative results.

Model	Mean IoU. (%)	Loc Acc. (%)
ResNet-101	38.7	52.4
ConvNeXt-S	41.2	55.8
+DKBlock	40.9	54.6
Dilate-B	45.6	59.6
Swin-S	45.6	59.7
ViT-Base	42.5	52.1
Ours	64.2	73.5

## Data Availability

The datasets used in this study are publicly available, including ETH Food-101, Vireo-Food-172, and ISIA Food-500.
